# *Caenorhabditis elegans* Genomic Response to Soil Bacteria Predicts Environment-Specific Genetic Effects on Life History Traits

**DOI:** 10.1371/journal.pgen.1000503

**Published:** 2009-06-05

**Authors:** Joseph D. Coolon, Kenneth L. Jones, Timothy C. Todd, Bryanua C. Carr, Michael A. Herman

**Affiliations:** 1 Ecological Genomics Institute, Kansas State University, Manhattan, Kansas, United States of America; 2 Division of Biology, Kansas State University, Manhattan, Kansas, United States of America; 3 Department of Plant Pathology, Kansas State University, Manhattan, Kansas, United States of America; Stanford University Medical Center, United States of America

## Abstract

With the post-genomic era came a dramatic increase in high-throughput technologies, of which transcriptional profiling by microarrays was one of the most popular. One application of this technology is to identify genes that are differentially expressed in response to different environmental conditions. These experiments are constructed under the assumption that the differentially expressed genes are functionally important in the environment where they are induced. However, whether differential expression is predictive of functional importance has yet to be tested. Here we have addressed this expectation by employing *Caenorhabditis elegans* as a model for the interaction of native soil nematode taxa and soil bacteria. Using transcriptional profiling, we identified candidate genes regulated in response to different bacteria isolated in association with grassland nematodes or from grassland soils. Many of the regulated candidate genes are predicted to affect metabolism and innate immunity suggesting similar genes could influence nematode community dynamics in natural systems. Using mutations that inactivate 21 of the identified genes, we showed that most contribute to lifespan and/or fitness in a given bacterial environment. Although these bacteria may not be natural food sources for *C. elegans*, we show that changes in food source, as can occur in environmental disturbance, can have a large effect on gene expression, with important consequences for fitness. Moreover, we used regression analysis to demonstrate that for many genes the degree of differential gene expression between two bacterial environments predicted the magnitude of the effect of the loss of gene function on life history traits in those environments.

## Authors Summary

Transcriptional profiling is often used to identify genes that are differentially regulated in response to different environments. These experiments assume that genes differentially expressed in response to different environments are functionally important and, furthermore, that the degree of differential gene expression is predictive of the magnitude of functional importance. In genetic experiments, function is inferred from analyzing the phenotypes of removing, reducing or altering gene function. However, to date, there has not been a specific test of how well the degree of differential gene expression between two (or more) environments is predictive of gene function. Here we identified *C. elegans* genes that were differentially expressed in response to different bacterial environments and determined the phenotypic differences of life history traits between these environments using mutant strains that compromised gene function. We found that differential gene expression is indeed predictive of functional importance of the identified genes in different environments. This observation has important implications for interpreting the results of transcriptional profiling experiments of populations of organisms in their native environments, where in many cases the genetic tools to disrupt gene function have not yet been fully developed or interfering with gene functions in nature may not be feasible.

## Introduction

We are interested in understanding the genetic mechanisms that underlie organismal responses to their environment, especially in light of human induced environmental change. To begin to address this challenge have chosen to model the interaction of soil nematodes and their environment in the laboratory using an ecological genomic approach. Nematodes play key roles in many ecosystems including nutrient cycling, turnover of microbial biomass [1] and decomposition of soil organic matter [2],[3]. In fact, bacterial-feeding soil nematodes are among the most abundant invertebrates in soils and are important members of grassland belowground food webs [4]. In addition, many bacterial-feeding taxa have been shown to be among the most responsive of nematode trophic guilds to various disturbance regimes [5]–[7]. These responses include shifts in the species composition of bacterial-feeding nematode assemblages, resulting in altered community structure and, presumably, function. For example the increased relative abundance of opportunistic Rhabditidae species is characteristic of a response to resource pulses caused by disturbance or changing land management practices [1],[8]. The effects of disturbance, can be direct, such as changes in chemical and physical properties of the soil that impact nematode movement or life history, or indirect, such as changes in other biotic components (e.g., food source) that affect the nematode community. Here we begin to address the genetic basis of one such indirect effect. Recent studies have demonstrated that the grassland soil bacterial (KLJ, JDC, MAH, unpublished) and bacterial-feeding nematode communities [5] on the Konza Prairie Biological Station responded to various disturbance treatments with species-specific responses, indicating that indirect causes through bottom-up effects of the responses of the bacterial-feeding nematode community are plausible. Other recent studies conducted in other ecosystem types have demonstrated microbial community responses to disturbances comparable to the bacterial community response we observed on Konza prairie. For example, tilling for agriculture [9],[10], burning of aboveground biomass [11]–[13] and the addition of nitrogen amendments [14]–[16] can cause changes in the relative abundance of bacterial species and microbial community diversity. Thus, the differential bacterial community response to perturbation, in conjunction with nematode food preference [17] and/or pathogenic interactions [18] with bacteria could drive the observed changes in nematode community structure. Therefore, we have focused on the genomic responses of microbial-feeding nematodes to the possible changes within the grassland microbial environment. We hypothesize that an examination of the genomic response of nematodes to different bacterial environments may reveal the genetic basis of the observed nematode community response.

We are interested in the genetic responses of native ecologically relevant nematodes that do not have well developed genetic and genomic resources and thus are not tractable for functional studies. Specifically, we have identified several members of the *Rhabditididae* (*Mesorhabditis*, *Oscheius* and *Pellioditis*) and *Cephalobidae* (*Acrobeloides* and *Acroboles*) families whose relative abundance is altered in response to nutrient additions. Thus, we have turned to a laboratory modeling approach using a related, genetically tractable, bacterial-feeding soil nematode to identify conserved candidate genes. Many groups have analyzed the transcriptional response of the genetically tractable nematode *Caenorhabditis elegans* to various, usually medically significant, bacteria [19]–[22], in order to model human innate immunity [18],[23]. While the high degree of evolutionary conservation allows *C. elegans* to be a good model for human-bacteria interactions, it may be an even better model for bacterial-feeding nematode responses to bacteria. Although *C. elegans* is not found in abundance in the grassland soils under study [5], it is related to many of the relevant nematode taxa of interest. Therefore, we exposed *C. elegans* to different grassland soil bacteria and used transcriptional profiling to identify differentially expressed genes. We determined the functional significance of a subset of the differentially expressed genes by measuring fitness and lifespan of mutant nematodes in the various bacterial environments. Our results demonstrate that the functions of many of the genes specifically induced in response to different bacteria contribute to nematode fitness and lifespan in those bacterial environments. Furthermore, for specific genes, the extent of differential gene expression between bacterial environments was correlated with the degree of the effect of mutations in those genes on life history traits in those environments. Thus we propose that examination of differential gene expression in different environments allows for prediction of degree of mutational effects of those genes in those environments. Thus, here we show the first evidence, to our knowledge, that there is indeed good predictive power for the effects of mutant phenotypes in an environment-specific manner, suggesting that the relative level of transcription can be informative about the relative contributions to function, at least for life history traits. Additionally, the examination of *C. elegans* gene function in new environments has uncovered new phenotypes for previously studied genes as well as genes that had not been shown to have obvious phenotypes under standard laboratory conditions, perhaps adding to our understanding of the *C. elegans* genome.

## Results

### *C. elegans* response to soil bacteria

We are interested in understanding naturally occurring nematode-bacterial interactions of native soil nematodes. Since these nematodes do not have well characterized genomes or genetic tools, we have used *C. elegans* as a model to discover conserved genes involved in these interactions. For this purpose, we isolated bacteria from grassland prairie soils at the Konza Prairie Biological Station. Although *C. elegans* has not been found at the Konza prairie, related nematodes from the same family (Rhabditidae) are found there, thus it should be a suitable model nematode. *Micrococcus luteus* was the most abundant bacterial species in the nutrient amendment plots (supplemented annually with 10 g/m^2^ ammonium nitrate for 21 years) that was culturable on nematode growth media (NGM) plates (data not shown). Nematode growth media was used for bacterial isolation, as growth on NGM was a requirement of the experiment. *Bacillus megaterium* and *Pseudomonas* sp. were isolated in association with Rhabditid nematodes from Konza prairie soils (*Oscheius* sp. and *Pellioditis* sp. respectively) [5],[24]. Bacteria were isolated by extracting nematodes from the soil followed by thorough washing to remove bacteria weakly associated with the nematode cuticle. Nematodes were then placed on NGM plates and allowed to defecate surviving ingested bacteria. Although, this method of bacterial isolation makes it likely that bacteria came from nematode intestines, we cannot rule out that bacteria were associated with native nematode cuticles. Thus, these were termed nematode associated bacteria. The *Pseudomonas* sp. we isolated was most similar to *Pseudomonas fluorescens* with 98% sequence identity (Ribosomal Database Project) in the 16S rDNA sequence (See Methods).

**Figure 1:**
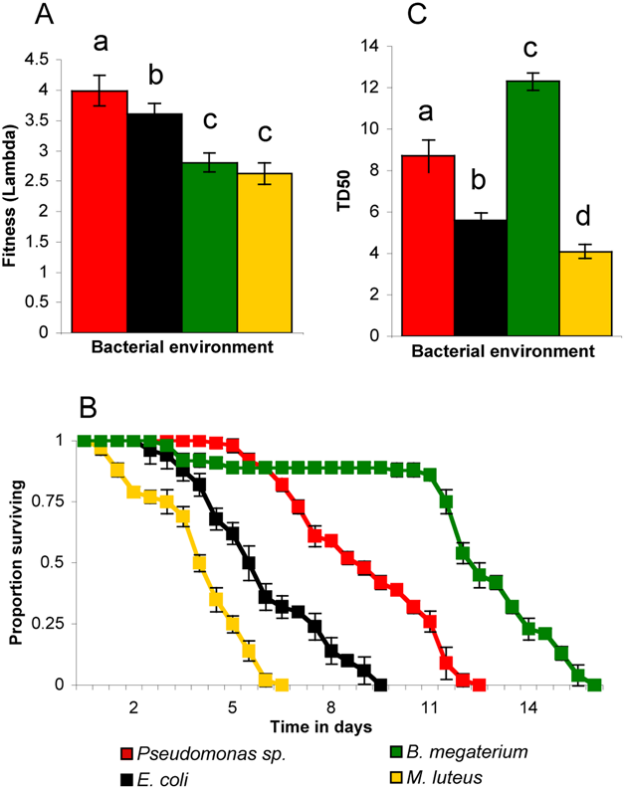
Effects of bacterial environment on wild-type life history traits. (A) Absolute fitness (λ) values for wild type (N2) in each bacterial environment. (B) Survivorship curves showing the proportion of the starting population surviving at different times in each bacterial environment. Letters over error bars indicate groups with significantly different means as determined by ANOVA. (C) Time to death for 50% of the individuals in a population (TD_50_) is shown for N2 grown in each bacterial environment. Standard error is indicated with error bars.

Wild-type *C. elegans* (N2) was grown on the three prairie bacterial species as well as *E. coli* (OP50) which served as a control, as it is the typical laboratory diet for *C. elegans* [25]. The different bacteria served as food sources for *C. elegans* as well as the immediate environment during growth as the culture plates contained bacterial lawns. Therefore, the effects of the external features of the different bacterial lawns (*e.g.* oxygen concentration in the bacterial lawn, bacterial viscosity and potential bacterial secretions) on nematode physiology could not be distinguished from the effects of ingestion of the bacteria and will hereafter be collectively referred to as ‘bacterial environment’. To get a more accurate estimate of the effects of different bacterial environments on nematode fitness, we used life tables to estimate absolute fitness (λ), which accounts for age specific fecundity (m_x_) and survival (l_x_), as well as generation time (T) [26] and is subsequently more comprehensive than brood size alone (see Methods). The absolute fitness of wild-type animals differed significantly in the different bacterial environments. Animals displayed the highest fitness when grown on *Pseudomonas* sp. (λ = 3.99), which was significantly greater (*P* = 0.021) than when grown on *E. coli* (λ = 3.60), *B. megaterium* (λ = 2.81, *P*<0.0001) and *M. luteus* (λ = 2.63, *P*<0.0001). Fitness of wild-type animals on *E. coli* was also significantly higher than on either *B. megaterium* (*P* = 0.027) or *M. luteus* (*P* = 0.027; Figure 1A, Table 1). It is interesting to note that the only previous study to use life tables to calculate fitness in *C. elegans* [27] found highly similar values (λ = 3.85, with growth on *E. coli* OP50).

**Table 1:** Biological validation of identified genes.

Gene	*E. coli* (OP50)		*M. luteus*		*Pseudomonas* sp.		*B. megaterium*	
	λ	TD_50_	λ	TD_50_	λ	TD_50_	λ	TD_50_
wt	3.60(0.19)	5.6(0.22)	2.63(0.18)	4.1(0.22)	3.99(0.25)	8.7(0.27)	2.81(0.16)	12.3(0.27)
*acdh-1*	2.99(0.03)^−^	5.0(0.35)^−^	2.54(0.25)	5.0(0.35)^+^	3.78(0.74)	5.5(0.79)^−^	3.01(0.37)	10.4(0.42)^−^
*C23H5.8*	2.72(0.03)^−^	7.8(0.57)^+^	2.42(0.04)^−^	3.6(0.42)^−^	3.07(0.02)^−^	6.0(0.79)^−^	3.30(0.04)^+^	8.9(0.74)^−^
*cey-2*	3.08(0.04)^−^	6.1(0.42)	2.11(0.06)^−^	3.5(0.35)^−^	2.83(0.03)^−^	7.5(0.61)^−^	2.79(0.01)	7.0(0.35)^−^
*cey-4*	3.51(0.13)	5.6(0.42)	2.84(0.06)^+^	3.6(0.42)^−^	3.57(0.07)^−^	5.9(0.22)^−^	2.95(0.02)	3.7(0.27)^−^
*cpi-1*	3.25(0.15)^−^	7.6(0.22)^+^	3.01(1.17)	4.4(0.22)	3.65(0.43)	6.6(0.42)^−^	3.19(0.41)	12.4(0.42)
*ctl-1*	2.91(0.07)^−^	6.2(0.84)	2.53(0.07)	4.8(0.29)^+^	2.77(0.18)^−^	3.9(0.42)	2.29(0.07)^−^	8.5(0.35)^−^
*cyp-37A1*	3.59(0.08)	8.0(0.50)^+^	2.37(0.06)^−^	4.4(0.42)	3.64(0.03)^−^	8.5(0.35)^−^	2.85(0.04)	9.5(0.50)^−^
*dhs-28*	2.23(0.18)^−^	6.7(0.27)^+^	2.01(0.21)^−^	3.6(0.22)^−^	2.43(0.14)^−^	7.3(0.27)^−^	1.86(0.27)^−^	10.2(0.76)^−^
*dpy-14*	1.89(0.44)^−^	2.4(0.22)^−^	1.60(0.07)^−^	2.1(0.22)^−^	1.85(0.17)^−^	3.1(0.42)^−^	0.96(0.02)^−^	4.1(0.42)^−^
*dpy-17*	2.84(0.52)^−^	4.0(0.35)^−^	2.70(0.34)	3.1(0.42)^−^	3.20(0.45)^−^	3.0(0.35)^−^	2.69(0.80)	12.3(0.57)
*elo-5*	4.11(0.07)^+^	5.5(0.35)	3.02(0.10)^+^	2.6(0.42)^−^	4.07(0.12)	5.0(0.50)^−^	4.18(0.05)^+^	9.5(0.35)^−^
*F55F3.3*	3.53(0.15)	3.1(0.55)^−^	2.25(0.14)^−^	2.6(0.55)^−^	2.24(0.07)^−^	5.0(0.35)^−^	2.06(0.07)^−^	5.5(0.35)^−^
*fat-2*	3.27(0.13)^−^	9.9(0.82)^+^	2.97(0.04)^+^	8.5(0.35)^+^	4.23(0.04)	11.4(0.74)^+^	3.18(0.09)^+^	13.7(1.15)^+^
*gei-7*	3.52(0.25)	5.7(0.27)	2.73(0.12)	4.5(0.00)^+^	3.77(0.26)	7.6(0.22)^−^	3.27(0.48)	14.3(0.27)^+^
*gld-1*	3.15(0.13)^−^	5.6(0.22)	2.51(0.28)	3.5(0.35)^−^	3.53(0.06)^−^	4.3(0.57)^−^	2.78(0.04)	5.5(0.35)^−^
*hsp-12.6*	3.10(0.08)^−^	5.7(0.45)	2.50(0.18)	3.7(0.27)^−^	3.72(0.14)	6.6(1.29)^−^	3.00(0.08)^+^	9.5(1.00)^−^
*mtl-2*	3.77(0.17)	6.1(0.22)^+^	3.02(0.23)^+^	5.2(0.27)^+^	4.09(0.28)	8.0(0.35)^−^	3.75(0.40)^+^	13.8(0.27)^+^
*pab-2*	4.14(0.06)^+^	6.6(0.42)^+^	2.72(0.47)	5.4(0.42)^+^	4.29(0.24)	7.7(0.57)^−^	3.20(0.11)^+^	8.9(0.74)^−^
*rol-6*	2.82(0.22)^−^	3.1(0.82)^−^	2.28(0.11)^−^	2.9(0.22)^−^	3.11(0.09)^−^	7.7(0.45)^−^	2.56(0.04)^−^	10.2(0.76)^−^
*sqt-2*	2.97(0.01)^−^	6.9(0.42)^+^	2.69(0.06)	3.7(0.57)	3.72(0.06)^−^	4.2(0.57)^−^	3.39(0.47)^+^	7.2(1.35)^−^
*Y57A10C.6*	3.37(0.18)	6.3(0.45)^+^	2.09(0.09)^−^	4.5(0.00)^+^	3.41(0.33)^−^	8.2(0.57)	2.62(0.23)	15.0(0.35)^+^

*Wild-type* (N2) and mutant *C. elegans* strains were grown on the four bacterial isolates and absolute fitness (λ) and time to death for 50% of the individuals in a population (TD_50_ in days) was measured. *P*-values are shown for contrasts between environments within strain for λ and TD_50_. Standard error (s.e.m.) is given in parenthesis. + indicates a significant (*P*<0.05) increase relative to wild type and − indicates a significant (*P*<0.05) decrease of the mutant relative to *wild-type*.

Lifespan is another important aspect of nematode demography. Lifespan is measured here as time to death for 50% of a population (TD_50_) [28],[29] using survivorship curves (Figure 1B and Figure S1A, S1B, S1C, S1D). Lifespan is a complex trait; with the pathogenicity of *C. elegans* food sources being a major component, as it has been suggested that bacterial colonization and resultant tissue damage is the major cause of nematode death even on the standard *E. coli* strain OP50 [30],[31]. Van Voorhies *et al.* showed that the substrate in which lifespan is measured is important with wild-type *C. elegans* lifespan in soil being much shorter than lifespan when grown on agar plates [32]. However, in order to simply investigate the effects of bacterial environment we have chosen to use the more controlled agar plate substrate for *C. elegans* growth. Wild-type animals had lower TD_50_ values (i.e. died more quickly) when grown on *M. luteus* (TD_50_ = 4.1) than during growth on *E. coli* (TD_50_ = 5.6), while growth on both *Pseudomonas* sp. (TD_50_ = 8.7) and *B. megaterium* (TD_50_ = 12.3) increased lifespan with all pair-wise comparisons of the four bacterial environments significant (*P*<0.0001) (Figure 1C, Table 1). The extended lifespan in the *B. megaterium* environment is not likely a consequence of starvation, as generation time (thus larval developmental rate) is not severely altered as would be expected of worms under caloric restriction [33],[34] (Table S1). Wild-type lifespan on *E. coli* OP50 was only 5.6 days, this is in line with some studies [35] and lower than in others [36] possibly illustrating lab to lab differences in OP50 strains.

**Figure 2:**
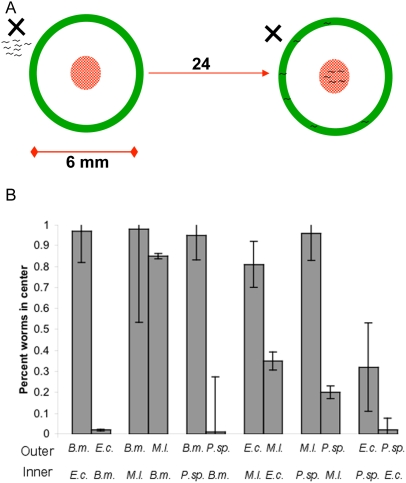
Food preference. (A) Food preferences of wild-type animals were measured in a biased choice assay modified from Shtonda and Avery (2006). Bacteria were arrayed on an agar plate as shown. Synchronized L1 larvae were placed outside the outer circle (indicated by the X) and the fraction in the center bacterial type was determined after 24 hours. (B) Fraction of nematodes in the center bacterial type is shown for all pair-wise comparisons, and reciprocal comparisons were used for *C. elegans* food preference. Standard error for each mean is indicated with error bars. The bacteria listed under each bar were compared and are either outer (outer ring) or inner (inner circle) and B.m. = *B. megaterium*, M.l. = *M. luteus*, E.c. = *E. coli*, P.sp. = *Pseudomonas* sp.

To further characterize wild-type *C. elegans* response to the bacterial isolates we conducted food preference tests. In a previous study [17], it was found that food choice was comprised of more than chemotaxis and there was a dynamic of both food seeking and food leaving behavior whereby *C. elegans* seeks out higher quality food sources and leaves behind hard to eat bacterial types. Additionally, the same study reported that while *C. elegans* had little chemoattraction to various tested bacterial species, there was obvious food preference measured by a biased choice assay. As we were interested in food preference, we therefore chose to use the biased choice assay (Figure 2A) rather than chemotaxis assays [17] and we determined food preferences for all pair-wise combinations of bacterial isolates (Figure 2B). We observed a hierarchy of food preferences: *Pseudomonas* sp. was most preferred, closely followed by *E. coli*, both of which were preferred over *B. megaterium*, followed by *M. luteus*. Interestingly, this hierarchy mirrored the observed trend for fitness in the different bacterial environments (Figure 1A), with *C. elegans* preferring *Pseudomonas* sp. on which it was most fit, followed by *E. coli*, *B. megaterium*, and *M. luteus*, respectively. Thus *C. elegans* food preference appears to correlate with fitness, with bacterial environments on which worms were most fit being preferred.

### Genomic transcriptional response

Transcriptional responses of wild-type *C. elegans* adults were assayed after growth on each of the four bacteria: *E. coli*, *M. luteus*, *Pseudomonas* sp., or *B. megaterium*. While dauer formation is an important aspect of the *C. elegans* life cycle, we have not observed dauer formation in all the native nematodes species that we are modeling with *C. elegans*. Therefore, young adult animals were analyzed; this also reduced the possibility that age differences confounded gene expression responses to the different bacterial environments (Figure S2). A total of 372 genes were shown to be differentially expressed and statistically significant with multiple testing correction (q<0.01, [37]) across all pair-wise comparisons. Of these, 366 were differentially expressed greater than two-fold and six less than two-fold, illustrating the high power associated with six biological replicates. The 372 genes correspond to a total of 204 unique genes identified across all comparisons (e.g., some genes had significant interactions with multiple bacteria; Tables 2, Table S2). Microarray expression levels of ten genes were verified using quantitative Polymerase Chain Reaction (qPCR) and found to be comparable to the microarray results, indicating that on average the expression differences revealed in the microarray analyses were reliable (Table S3).

**Table 2:** Identification of differentially expressed genes.

Comparison	No. of genes
*B. megaterium* vs. *E. coli* (OP50)	55
*B. megaterium* vs. *M. luteus*	25
*B. megaterium* vs. *Pseudomonas* sp.	81
*E. coli* (OP50) vs. *M. luteus*	41
*E. coli* (OP50) vs. *Pseudomonas* sp.	62
*M. luteus* vs. *Pseudomonas* sp.	108
TOTAL	372
UNIQUE	204

Statistical analysis was performed using a mixed model ANOVA. False discovery rate (q<0.01) was used to determine significance thresholds for identification of differentially expressed genes. Given the overlap of differentially expressed genes identified between the six comparisons, the number of unique genes identified is also included.

**Figure 3:**
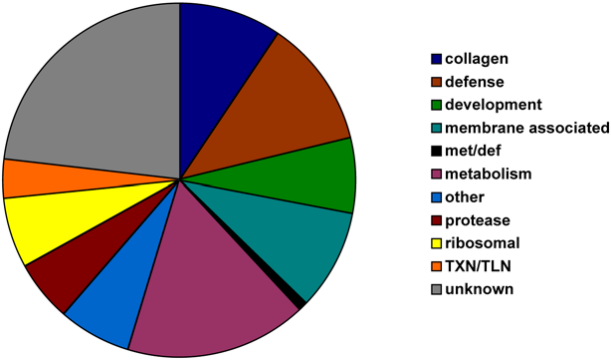
Gene Ontology terms for the 204 identified differentially expressed genes. GO terms were amended with recently published information and used to categorize the identified differentially expressed genes. Clustering was done manually by grouping GO terms of similar function. TXN/TLN = transcription/translation-related, met/def = roles in both metabolism and defense. Other = groups represented by two or less members.

Gene Ontology (GO) terms for the identified genes were used to group genes by similar function (See Methods). Metabolism genes were highly represented (16.6%) as expected. Interestingly, genes previously implicated in innate immunity were found in all six comparisons (11.6%). Specifically, we found 20 defense genes upregulated in response to *M. luteus*, 12 in response to *E. coli*, 14 in response to *B. megaterium* and two in response to *Pseudomonas sp.* That we found defense genes upregulated in response to the latter two bacteria was unexpected, as they cause an increase in lifespan relative to *E. coli* (Figure 1B, Table 1). Surprisingly, 9.5% of identified genes were involved in cuticle biosynthesis or collagens and 9.0% were membrane associated. Other groups found to make up smaller proportions were developmental (7%), ribosomal (6.5%), proteases (5.5%), and gene expression (3.5%). Finally, genes of unknown function made up the largest portion (23.1% of the total, Figure 3, Table S2), also as expected since one aim of this work was to determine functions for such genes.

We also mapped the identified genes to the *C. elegans* co-expressed gene mountains as an additional approach to determine functional groups and found similar over-represented groups, as quantified by the representation factor (RF, Table S4, [38]. Thirty-two (RF = 3.4, *P* = 7.9e-05) of the identified genes mapped to mount 8, which is enriched with genes associated with mitosis as well as genes previously implicated in innate immunity. Mount 19, which is comprised predominately of genes involved in glycolysis, contained 14/204 (RF = 6.4, *P* = 4.2e-06) identified genes. Twenty-five of our identified genes were found in the mount 22, which represents genes involved in carbohydrate metabolism (RF = 14.3, *P* = 4.7e-20). Thus two different methods clustered our identified genes similarly, indicating an enrichment in genes for metabolic and defense mechanisms presumably for protection and nutrition.

### Biological validation of identified genes

We obtained all available viable non-sterile loss-of-function mutations for the 204 differentially expressed genes in our study, (21/204, or ∼10% of the total identified genes had available mutants) from the *Caenorhabditis* Genetics Center (CGC) and used them for biological validation of our microarray results (Table S5). We performed functional tests measuring multiple aspects of life history including brood size, generation time (Table S1, Figure S3), absolute fitness and lifespan (TD_50_) (Table 1, Figure S1) for all four bacterial environments. We found that many of the mutations had effects on life history traits and differed significantly from wild type in a given bacterial environment. While the majority of mutant strains tested had decreased fitness compared to *wild type* in each environment, surprisingly, a few mutant strains showed increased fitness when grown on *E. coli*, *M. luteus* and *Pseudomonas* sp. (Table 1 and Table 3). Interestingly, more mutant strains had increased fitness in the *B. megaterium* environment as compared to *wild type* than had reduced fitness.

**Table 3:** Summary of mutant life history responses in each bacterial environment.

		*Ec*	*Ml*	*Psp*	*Bm*
Fitness (λ)	# up	2	4	0	7
	# down	13	8	13	5
	total	15	12	13	12
Lifespan (TD50)	# up	9	7	1	4
	# down	5	11	18	15
	total	14	18	19	19
Brood size	# up	0	1	0	5
	# down	8	4	10	5
	total	8	5	10	10
Generation time (T)	# up	9	5	8	6
	# down	2	3	1	9
	total	11	8	9	15

Mutant life history traits: Fitness, Lifespan, Brood size, and Generation Time were determined in each bacterial environment (Table 1, Table S1) and significant (ANOVA, *P*<0.05) relative differences (up or down) from wild type were determined for each mutant in each environment. For each trait, total is the number of mutants (from 21 tested) that differed significantly from wild type in each bacterial environment; # up is the number of mutants that had values greater than wild type and # down is the number of mutants that had values less than wild type. Note: *E* = *E. coli*, *M* = *M. luteus*, *P* = *Pseudomonas* sp., *B* = *B. megaterium*.

A similar trend was found for brood size as most mutant strains had reduced numbers of progeny in response to growth on the *E. coli*, *M. luteus*, and *Pseudomonas* sp., while growth on *B. megaterium* resulted in equal numbers of mutant strains that significantly increased and decreased brood size (Table S1, Table 3). Similarly, generation times were slower for most mutants in the same three bacterial environments and only in the *B. megaterium* environment were there more mutants with faster generation times (Table 3). Surprisingly, lifespan showed a different trend. Growth of mutant strains on *Pseudomonas* sp., *M. luteus* and *B. megaterium*, primarily caused reductions in lifespan, while growth of mutant strains on *E. coli* resulted in the majority having significantly increased lifespan. Overall, many of the mutational affects on life history were environment specific, demonstrating that transcriptional profiling identified genes of functional importance in each bacterial environment.

### Differential expression predicted genetic effects on life history traits

**Figure 4:**
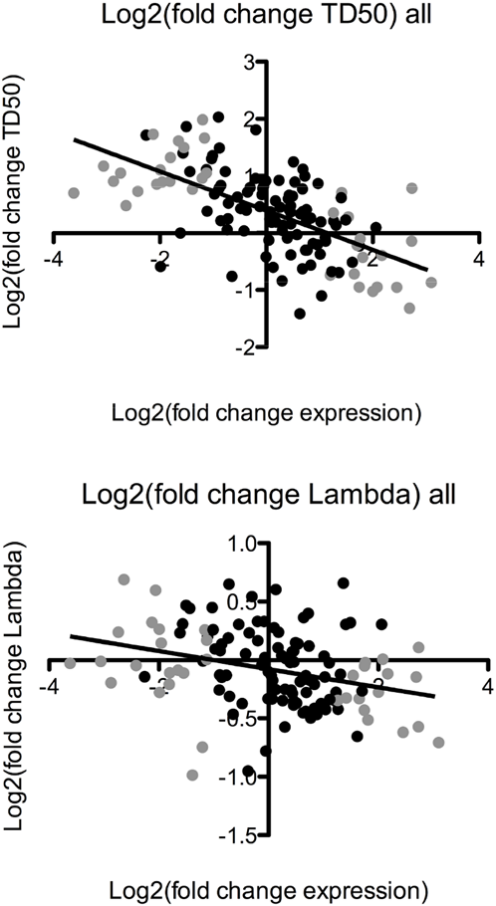
Regressions of life history on relative expression. Relative expression was used as a predictor for life history traits of mutants. Linear regressions were performed using Log_2_ transformed fold change in gene expression from microarray experiments as the independent variable and Log_2_ transformed fold change in (A) Lifespan (TD50) or (B) fitness (λ) in bacterial comparisons of mutant life history traits. Grey circles are those instances of genes identified as significantly (q<0.01) differentially expressed, and black circles are from the same 21 genes but were the instances where significant differential expression was not observed.

We next tested whether differential gene expression between environments had predictive power for the mutational effects on life history traits (lifespan, fitness, generation time and brood size) in those environments. We tested the correlation between the change in relative gene expression and the phenotypic difference in life history traits of a strain containing a mutation in a given gene (Figure 4). We predicted that most genes that were up-regulated in an environment would positively regulate a particular life history trait, such that loss or reduction of that gene function would cause a reduction in fitness or lifespan (or brood size and a possible increase in generation time) in that environment. Therefore, our *a priori* expectation would be that data points would fall in the lower right and the upper left quadrants for lifespan, fitness and brood size, and the exact opposite for generation time. Indeed, we found that there is a correlation (r = −0.62) between mutant lifespan [Log_2_(fold change TD_50_)] and differential expression of genes in comparisons of bacterial environments (Table 4). The slope for the regression was negative, as expected given our prediction that up-regulated genes positively regulate lifespan, and the slope was found to significantly non-zero (p<0.0001; Table 4). It was striking that a correlation was observed for the 21 genes across all six comparisons (126 total comparisons), as most involved gene-by-bacterial comparisons for which we did not observe significant differential expression. In fact independent correlations of only those gene-by-bacteria comparisons that had significant differential expression (q<0.01) were more strongly correlated (r = −0.73), while those that did not have significant differential expression (q>0.01) were less correlated (r = −0.53, Table 4).

**Table 4:** Correlation of gene expression and phenotypic effects.

Life history	Data set	r	Slope	*P*-value
Log_2_(fold change TD_50_)	All	−0.62	−0.34	<0.0001
	q<0.01	−0.73	−0.32	<0.0001
	NS	−0.53	−0.41	<0.0001
Log_2_(fold change lambda)	All	−0.31	−0.077	0.0004
	q<0.01	−0.44	−0.074	0.0058
	NS	−0.26	−0.099	0.0141

Correlational analysis was performed using Log_2_ transformed fold change values from the transcriptional profiling experiments as the independent variable and Log_2_ transformed fold change in lifespan (TD_50_) or fitness (λ) as the dependent variable. Pearson correlation coefficients (r) and slope values for the best-fit lines are indicated. Additionally, *P*-values corresponding to tests of slope≠0 are shown. NS are genes not significantly differentially expressed (q>0.01) in transcriptional profiling.

The same analysis performed on fitness (λ) revealed a reduced correlation between relative expression and fitness of mutants compared to that of lifespan. Overall, the correlation coefficient for the relationship between differential expression fitness was r = −0.31 (Table 4). The slope of the best-fit line was −0.077 and significantly non-zero (*P*<0.0004, Table 4). The correlation for the genes found to have significant differential expression (q<0.01) was again better than the complete data set (r = −0.44) and reduced in the gene by bacteria comparisons not found to be significantly differentially expressed (r = −0.26, Table 4).

Correlations were also performed on generation time and brood size, both constituents of fitness (λ). The correlation coefficient for generation time and fold change in gene expression (r = −0.057) indicates poor fit for the model, while the slope was significant (Figure S3, Table S4). Interestingly, there was a much stronger relationship between brood size and differential expression (r = 0.34), which was similar to that of fitness (Table S4) yet still reduced compared to lifespan. However, the regression best-fit line for brood size on differential expression had a non-significant slope (Figure S3, Table S4). Taken together, these tests suggest that while both brood size and generation time are important factors in the relationship between fitness and differential expression, brood size might have a larger contribution.

To illustrate the relationship between the level of gene expression and the degree of phenotypic effect, we consider two examples. The first is *hsp-12.6* which encodes a heat-shock protein [39] and was found to be up-regulated 2.4-fold when wild-type worms were grown on *E. coli* as compared to growth on *B. megaterium* (Table S2). We found that *hsp-12.6* mutants had a 15% proportional reduction in fitness as compared to wild type when the mutant was grown on *E. coli* (Figure S4A) which is significantly different (*p*<0.001) from that observed on *B. megaterium*. Not only is this difference significant, but the fitness of *hsp-12.6* mutants was also significantly increased relative to wild type when grown on *B. megaterium* (Table 1). While the exact role *hsp-12.6* plays in these particular interactions of *C. elegans* with bacteria remains to be elucidated, our results suggest that there was a cost associated with the expression of *hsp-12.6* in an environment in which it was not needed and a detriment to loss of function in an environment in which it is needed.

Another example of the relationship between level of gene expression and degree of phenotypic effects was the effect of a *rol-6* recessive loss-of-function mutation on lifespan. Transcriptional profiling showed that *rol-6* was up-regulated 2.7-fold in the *E. coli* versus *Pseudomonas* sp. comparison, which is surprising as *rol-6* encodes a cuticular collagen not previously implicated in response to bacteria. However we did observe that genes involved in cuticle formation and function, which includes *rol-6*, were among the most over-represented groups of genes identified, suggesting modulations in cuticle function could be common to *C. elegans* interactions with bacteria. Thus, our prediction was that loss of *rol-6* function would decrease lifespan in an environment-specific manner, which was what we observed. When the *rol-6* mutant strain was grown on *E. coli* there was a 45% proportional reduction in TD_50_ compared to the 11% reduction observed on *Pseudomonas* sp. and this difference was significant (*p*<0.0001, Figure S4B). Interestingly, *rol-6* has been well characterized and extensively studied [40]–[43] for its role as a cuticular collagen and is needed for proper cuticle morphology; yet through use of alternate environments an additional role for *rol-6* function in defense was uncovered.

## Discussion

We characterized the effect of exposing *C. elegans* to different soil bacterial food sources/environments to model naturally occurring interactions that may be driving changes in the bactivorous nematode community in response to land use change in grasslands [5]. Although these specific bacterial environments might not be encountered by *C. elegans* in the wild, *C. elegans* is likely to encounter various related bacterial species in its natural environment and is an excellent model for understanding the responses of bactivorous soil nematodes to their bacterial environment. Using transcriptional profiling we set out discover genes that function in environmental interactions, specifically interactions with bacteria. We identified 204 genes that were significantly differentially expressed when adult worms were grown on different bacterial food sources/environments isolated from grassland soils. Most of the identified genes were characterized to be involved in metabolism, defense, cuticle biosynthesis, or were of unknown function (Figure 3). In addition, 46 genes without annotation were identified, which can now be further investigated and functions determined, helping to further our understanding of the *C. elegans* genome.

A unique aspect of this work is that we calculated fitness using life tables. To our knowledge this is the first use of such analyses to biologically validate candidate genes identified by transcriptional profiling. In addition, we showed a strong correlation between the changes in relative gene expression in comparisons of environments using transcriptional profiling and the phenotypic differences of life history traits when that gene's function was compromised. The best relationship we observed was for lifespan on differential expression, which seems logical given the environments used were bacterial food sources. The correlation of fitness on differential expression was not as high, however. While lifespan is a complex trait controlled by many genes [44], perhaps fitness is an even more inclusive and complex trait that may be controlled by the interaction of many more genes. If this were the case, we would expect the relationship between single mutant effects on fitness in different environments and differential expression between those environments to be more complex. We observed a strong correlation between gene expression and life history trait despite the multiple factors that might complicate the relationship including, genes involved in negative regulation, redundancy, effects of genetic network structure to name a few. This demonstrates the predictive power of transcriptional profiling, at least when used to investigate responses to different external stimuli. Interestingly, other studies that have used gene inactivation to biologically validate environmentally induced differential expression, investigating the responses of *C. elegans* to cadmium exposure and *D. melanogaster* to alcohol exposure [45],[46], have also found that a large proportion of genes are functionally important suggesting this could be a common feature to transcriptional regulatory networks that are involved in response to external stimuli [47]. Furthermore, we suggest that not only can transcriptional profiling be used to identify relevant candidate genes, but also the direction and magnitude of expected mutant phenotypes of those genes in response to different environments, ultimately demonstrating their functional importance.

In addition, we used new environments to identify phenotypes for genes of unknown function as well as to show new aspects of phenotypes for previously well-characterized genes. For example, we observed differential expression of *pab-2*, which encodes a poly-A binding protein, in our microarray experiments and also demonstrated that *pab-2* mutants had a significantly higher fitness (λ = 4.14) than wild type (λ = 3.60) when grown on *E. coli* (Table 1). This result is surprising as N2 was cultured on *E. coli* OP50 for decades (>1,000 generations) prior to being frozen [44]. It is likely that during this extended period of time that *wild type* worms became better adapted to life on *E. coli*, yet *pab-2* mutants had a longer lifespan (TD_50_ = 6.6 vs. 5.6, Table 1), larger brood size (300.2 vs. 290.8) and a faster generation time (4.01 days) than wild type (4.4 days, Table S1) when grown on *E. coli*. Interestingly, this mutant does not follow the trend postulated by Hodgkin and Barnes [48] that there would be a trade-off between developmental rate and brood size because of differences in resource allocation, as there does not appear to be a trade-off between developmental rate and brood size for *pab-2* mutant animals. Further investigation will be required to elucidate the mechanisms by which fitness is increased in *pab-2* mutant animals.

When the identified differentially expressed genes were grouped by similar function we found significant enrichment for genes encoding cuticular collagens. This enrichment was also recently found for genes differentially expressed in response to other bacterial species [36]. Wong *et al.* (2007) found that many *C. elegans* cuticular collagens were part of a shared response, indicating that they were regulated in response to multiple pathogens (*S. marcescens*, *E. faecalis*, *E. carotovora*, and *P. luminescens*), suggesting this may be a common response to pathogens. When the functions of the cuticular collagen genes were compromised we found that many had significant effects on lifespan in an environment-specific manner, suggesting this is not merely the consequence of a general “sick” phenotype associated with abnormal cuticle. Furthermore, as these collagen genes were identified through their differential expression in adult worms, differences in juvenile molting are not the cause of their differential expression. Taken together these data suggest that cuticle function may be complex and cuticle structure may be much more dynamic than previously thought, perhaps changing in response to environmental perturbations. It has been show that some bacteria species secrete extracellular proteases and this is an effective nematocide and virulence factor aiding in the pathogen-associated killing of nematode species [49]–[51]. These proteases act to degrade the cuticle of nematodes ultimately leading to their death. It is possible that *C. elegans* differentially expresses cuticular collagens in response to bacterial protease secretions in an attempt to repair or avoid their pathogenic effects, however further experiments will be need to investigate this hypothesis.

It was somewhat curious that we observed genes involved in innate immunity to be induced in response to *Pseudomonas sp.* and *B. megaterium* (Table S2). The response to *Pseudomonas sp.* was curious because we found that *wild type C. elegans* was most fit on this bacteria (Figure 1A) and others have found that *P. fluorescens*, which is similar to our isolate, does not affect *C. elegans* lifespan [52]. The response to *B. megaterium* was curious because we showed our isolate increased lifespan (Figure 1B) and others found another isolate did not affect lifespan [53]. One possible explanation of these results is that the *C. elegans* genome is poised to respond to these bacteria and that the induction of these genes successfully protects worms from these bacteria.

While the responses of some genes can be easily reconciled, the roles of others are more difficult to understand. At first glance it may be difficult to reconcile how *gld-1*, which functions to limit the proliferation of the gonad germ cells [54],[55], could play a role in the interactions with the environment. However, as *gld-1* regulates germ cell proliferation, it is well positioned to integrate signals from the intestine (and elsewhere) to control reproductive output. Specifically, we observed that *gld-1* was up-regulated in the *B. megaterium* vs. *Pseudomonas* sp. environmental comparison, suggesting that germline proliferation was inhibited in the presence of *B. megaterium*. Furthermore, *gld-1* mutants had a lower fitness in the *B. megaterium vs. Pseudomonas* sp. environment, suggesting the modulation of *gld-1* expression is functionally important. This may be an example of an organism reallocating energy from reproduction to other functions in response to environmental stresses or changes. Thus modulation of *gld-1* expression may allow for use of energy for functions other than reproduction including immune response. Interestingly, targets of the insulin signaling pathway (downstream of DAF-2/DAF-16), including innate immunity genes, have been shown to suppress *gld-1* induced tumors [56],[57], indicating that immune response through insulin signaling could influence reproductive output. Recently, the activities of specific developmental signaling pathways involved in vulval development have also been shown to be modulated in response to environmental perturbations. In particular, the Notch pathway, which functions with *gld-1* to control germ line proliferation, appears to be sensitive to perturbations in food availability [58] . This suggests that while development is robust in response to changes in the environment, slight modulations in processes that affect fitness traits do occur.

We also found that the hierarchy of food preference for the four bacterial isolates mirrored the trend observed for fitness of wild-type *C. elegans* in the different bacterial environments. This suggests that *C. elegans* prefers the environment in which it will be most fit. This observation is similar to that of Shtonda and Avery [17] except that their “food quality” measure only took into account developmental rate whereas the measures of fitness shown here also include age-specific fecundity and survival. It has also been suggested that bacterial size is an important determinant of food “quality” with bacteria of smaller diameter having higher quality [59], however we observed that the bacteria with the smallest diameter (*M. luteus*) resulted in *C. elegans* having the lowest fitness (Table S1) whereas *B. megaterium*, the largest bacteria tested (data not shown), did not have the dramatic effect previously shown [59]. Recently Rae *et al.*, (2008) reported that *Pristionchus pacificus*, another bacterial-feeding nematode associated with scarab beetles, displays differential attractions and susceptibilities to the various bacteria isolated in association with it. The authors suggest that *P. pacificus* discriminates among bacteria in its environment to maximize reproductive success [60]. Interestingly, *P. pacificus* has also been found in Konza prairie soils (B. Darby and MAH, unpublished). Food preference could therefore contribute to the mechanism driving observed nematode community structure in grassland soils, as we have recently found that perturbations that mimic disturbances caused by land-use change not only alter soil nematode communities [5] but also the soil bacterial community (KLJ, JDC and MAH unpublished) on Konza prairie. We have also observed that Konza soil nematodes differ in their susceptibility to the different bacteria tested here in terms of infection/colonization (JDC and MAH unpublished data), thus pathogenicity may also contribute to soil nematode community structure. Taken together our data suggest that the expression of metabolism and defense functions may in part drive nematode community dynamics in grassland soil systems through interactions with their bacterial environment. The results from this study suggest that the application of transcriptional profiling to native grassland nematode populations will help identify the functionally important gene functions involved in these interactions.

## Materials and Methods

### *C. elegans* and bacteria strains and maintenance

The following loss-of-function mutants were used: *cpi-1*(*ok1213*), *dpy-17*(*e1295*), *gei-7*(*ok531*), *mtl-2*(*gk125*), *dhs-28*(*ok450*), *Y57A10C.6*(*ok693*), *acdh-1*(*ok1489*), *rol-6*(*e187*), *ctl-1*(*ok1242*), *dpy-14*(*e188*), *fat-2*(*ok873*), *gld-1*(*op236*), *hsp-12.6*(*gk156*), *cey-2*(*ok902*), *cey-4*(*ok858*), *cyp-37A1*(*ok673*), *elo-5*(*gk182*), *pab-2*(*ok1851*), *sqt-2*(*sc108*), *F55F3.3*(*ok1758*), *C23H5.8*(*ok651*). Growth and maintenance conditions were as described [25],[61]. Use of native soil bacteria was as for *E. coli* (OP50). Bacterial isolate 16S rDNA was sequenced to identify species and sequence is available at NCBI's GenBank Database (accession numbers): *Micrococcus luteus* (EU704697), *Bacillus megaterium* (EU704698), *Pseudomonas* sp. (EU704696).

### Food preference and pathogenicity assays

Biased choice and lifespan assays were performed as previously described [17],[28],[52]. All pathogenicity tests were conducted in at least ten replicate experiments.

### Life table analysis

Demographic measures were collected on individual worms in the four bacterial environments. From life history measures including age specific reproduction and survival, life tables were used to calculate generation time, intrinsic growth rate and fitness calculated as lambda (λ). Mutant functional tests were performed by plating eggs onto the test bacteria and then placing progeny from this generation onto the test bacteria, one L4 hermaphrodite (P_0_ worm) per plate was incubated at 20°C with at least ten replicates per treatment per strain per environment. The original P_0_ worm was re-plated daily until death. Progeny per day was counted (age specific reproduction or m_x_). Survival of the P_0_ worm was monitored as well as the survival of all of the progeny from each reproductive period to determine age specific survival (l_x_). Using life table analysis, intrinsic growth rate (R_o_) was calculated as the sum of l_x_ times m_x_ (∑l_x_m_x_). Generation time (T) was calculated by (∑l_x_m_x_)/(∑xl_x_m_x_ where x = age class). Lambda was determined from R_o_ and T by calculating λ = e^(lnRo/T)^ , and λ was used as a measure of absolute fitness [26]. Replicate populations and subsequent life table calculations were used as replicates for statistical tests.

### Microarray hybridizations

Microarray hybridizations of *Caenorhabditis elegans* spotted oligonucleotide microarrays (Genome Sequencing Center at Washington University in St. Louis) were made using cDNA made from mRNA extracted from treated young adult *C. elegans* (N2). cDNA was made from extracted mRNA using Genisphere 3DNA Array350 kits according to manufacture recommendations (Genisphere Inc., Hatfield, Pennsylvania, USA). Microarray hybridizations were performed using a Tecan 400 Hybridization station (Tecan Inc., Zurich, Switzerland). Indirect labeling of cDNA was used to prevent hybridization bias associated with direct labeling procedures [62]. Hybridizations were carried out for 16 hours at 42° C according to manufacturer recommendations (Genisphere Inc. and Tecan Inc.). Hybridized arrays were scanned with an Axon GenePix 4000B (MDS Analytical Technologies, Toronto, Canada) and data was collected using GenePix 6.0 software (MDS Analytical Technologies). Gridding and preprocessing was done manually to remove bad spots and dye artifacts. Raw data files generated are MIAME compliant [63] and available at Gene Expression Omnibus (GEO) series accession number GSE15923.

### Microarray data analysis

All six pair-wise comparisons between treatment groups were made in a factorial design (Figure S1) to maximize the ability to detect differences between treatments [64]. Six biological replicates incorporating a dye swap for every other replication were performed to account for any potential dye bias associated with a particular fluorophore (i.e. Cy3 or Cy5, [62]). Data was analyzed as in Wolfinger *et al.* [65] using SAS statistical software (SAS Institute Inc., Cary, North Carolina, USA) using a two-step mixed model analysis of variance to account for all possible sources of variance. This two-step ANOVA was performed using the MIXED procedure in SAS, with the model for the first stage below and Y = background subtracted raw intensity from the raw data files generated by GenePix 6.0 (MDS Analytical Technologies).

Stage 1 model:
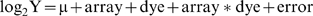
Where residuals, termed Relative Fluorescence Intensities (RFI) from stage 1 serve as the input for stage 2.

Stage 2 model:
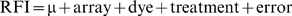



We used the false discovery rate (FDR) q to address the multiple testing problem [37]. q statistics were calculated in Q-VALUE and using the significance threshold q<0.01. We removed those genes that did not respond to bacterial environment in contrasts. Volcano plots were made in JMP 5.0 software (SAS Institute Inc., Cary, North Carolina, USA).An example of our SAS code can be found at (www.k-state.edu/hermanlab/SASCODE).

### Gene classification

Identified genes were assigned GO terms and manually grouped by similar function (Figure 1C, Table S2) incorporating when possible new annotations found in recent literature.

### Linear regressions and correlation analysis

Linear regressions were performed in GraphPad Prism 5 software using Log_2_(fold change expression) as the independent variable and Log_2_(fold change TD_50_), Log_2_(fold change lambda) Log_2_(fold change generation time) and Log_2_(fold change brood size) as the dependent variables. The linear regressions were performed on the 21 genes used in functional tests and for each gene, all 6 environmental comparisons for a total of 126 data points for each regression.

## Supporting Information

Table S1 Functional tests of brood size and generation time. Wild-type *C. elegans* and mutant strains were grown on the four bacterial environments and brood size and generation time were determined. Generation time was determined as T = (Σxl_x_m_x_)/(Σl_x_m_x_) (in days) using life tables. Standard error (s.e.m.) is given in parenthesis, and significant differences between mutant and wild-type is denoted by + or − (P<0.05) following values. Additionally + indicates an increase relative to wild type and a − indicates a decrease relative to *wild-type*.
(0.07 MB DOC)

Table S2 Significantly differentially expressed genes. Significantly differentially expressed genes are listed (by sequence name as they appear on the microarray .gal file) as well as the gene name, the directionality of the differential expression, the significance from statistical tests (see Methods), and the observed fold change. E = *E. coli*, M = *M. luteus*, P = *Pseudomonas* sp., B = *B. megaterium*. Additionally, the GO terms corresponding to the different genes have been summarized as used for Figure 3 (see Methods).
(0.08 MB XLS)

Table S3 qPCR validation results. We used quantitative reverse transcriptase real time PCR to validate the results of the microarray experiments. Three new biological replicates that were not used in the microarray experiments were used for validation. cDNA was synthesized for these RNA samples using a two-step iScript cDNASynthesis Kit (BioRad Laboratories) and these three replicate cDNA stocks for each bacterial environment were used for all subsequent tests. qPCR was performed with a Bio-Rad icycler (Bio-Rad Laboratories) using transcript specific primers. PCR primer sequences are available upon request. PCR reaction parameters were optimized as needed and housekeeping genes were used to standardize and calculate ΔCT values. From this ΔΔCT values were calculated and are shown in in the columns labeled qPCR. Microarray expression differences are shown for comparison. Melt curve analyses were performed to test for specific amplification. In all cases amplification was specific.
(0.06 MB DOC)

Table S4 Regression statistics for generation time and brood size. Regression analysis was performed using Log2 transformed fold change values from the transcriptional profiling experiments as the independent variable and Log2 transformed fold change in generation time or brood size as the dependent variable. Correlation coefficients (r) and slope values for the best-fit lines are indicated. Additionally, P-values corresponding to tests of slope≠0 are shown.
(0.04 MB DOC)

Table S5 Genes and alleles used for functional tests. List of 21 mutants used for functional tests and their predicted molecular functions and allele type are indicated.
(0.05 MB DOC)

Figure S1 Survivorship curves. Survivorship curves are shown for N2 and mutant strains across time for bacterial environments: (A) *B. megaterium* (B) *E. coli* OP50 (C) *M. luteus* (D) *Pseudomonas* sp.
(1.09 MB TIF)

Figure S2 Microarray experimental design and analysis. (A) The experimental design for the microarray comparisons made is shown. All pairwise comparisons of adult *C. elegans* in the four bacterial environments were made in a factorial design. Six biological replicates were used and dye-swaps were preformed every other replicate. (B) Volcano plots are shown for each microarray comparison. For each, −log_10_(p-value) is plotted on the Y-axis and log2(fold change) is plotted on the X-axis. Data points represent the response of all the genes present on the microarrays used, with each point representing a single gene. Points above the horizontal line are significant at the false discovery rate q<0.01. The two vertical lines show 2 fold up or down regulation in the treatment (relative to the second listed bacteria in graph titles).
(0.75 MB TIF)

Figure S3 Regression analysis of generation time and brood size. Linear Regressions were performed using Log2 transformed fold change in gene expression from microarray experiments as the independent variable and Log2 transformed fold change in (A) Generation time or (B) Brood size in bacterial comparisons of mutant life history traits.
(0.18 MB TIF)

Figure S4 (A) Proportional changes in *hsp-12.6* fitness were calculated as (μ_N2_−μ_hsp-12.6_)/μ_N2_ by bacterial environment to make relative to *wild type*. (B) Proportional changes in rol-6 longevity (measured as TD_50_) relative to wild type calculated as (μ_N2_−μ_rol- 6_)/μ_N2_ by bacterial environment. Letters indicate significantly different means (P>0.05 by ANOVA).
(0.13 MB TIF)
