# Supplementary material for: Caenorhabditis elegans Genomic Response to Soil Bacteria Predicts Environment-Specific Genetic Effects on Life History Traits
Source: PLoS Genet. 2009 Jun 5;5(6):e1000503. doi: 10.1371/journal.pgen.1000503 (PMC2684633; doi:10.1371/journal.pgen.1000503)
Supplement: Table S1 — Functional tests of brood size and generation time. Wild-type C. elegans and mutant strains were grown on the four bacterial environments and brood size and generation time were determined. Generation time was determined as T = (Σxlxmx)/(Σlxmx) (in days) using life tables. Standard error (s.e.m.) is given in parenthesis, and significant differences between mutant and wild-type is denoted by + or − (P<0.05) following values. Additionally + indicates an increase relative to wild type and a − indicates a decrease relative to wild-type. (0.07 MB DOC) [file pgen.1000503.s001.doc]

**Supporting Table 1**: Functional tests of brood size and generation time

|  | *E. coli* (OP50) | | *M. luteus* | | *Pseudomonas sp.* | | *B. megaterium* | |
| --- | --- | --- | --- | --- | --- | --- | --- | --- |
| Gene | Brood | T | Brood | T | Brood | T | Brood | T |
| *wt* | 290.80(60.87) | 4.40(0.19) | 171.00(70.12) | 5.15(0.36) | 307.00(49.16) | 4.11(0.25) | 210.83(57.22) | 5.10(0.20) |
| *acdh-1* | 283.20(43.15) | 5.14(0.16) | 172.67(53.43) | 5.46(0.55) | 290.83(104.7) | 4.32(0.39) | 184.83(38.73) | 4.80(0.59) |
| *C23H5.8* | 212.60(9.92)^-^ | 5.35(0.04)^+^ | 186.00(7.42) | 5.93(0.07)^+^ | 313.40(21.03) | 5.13(0.03)^+^ | 239.20(17.61) | 4.58(0.04)^-^ |
| *cey-2* | 348.20(18.03) | 5.20(0.07^+^ | 164.80(31.98) | 6.80(0.18)^+^ | 328.00(10.03) | 5.56(0.04)^+^ | 289.00(10.37)^+^ | 5.52(0.04)^+^ |
| *cey-4* | 313.80(16.48) | 4.58(0.14) | 208.60(3.29) | 5.12(0.09) | 356.60(12.26) | 4.62(0.08)^+^ | 363.40(19.73)^+^ | 5.45(0.05)^+^ |
| *cpi-1* | 260.20(46.09) | 4.71(0.13)^+^ | 122.00(101.4) | 4.21(0.49)^-^ | 228.17(27.38)^-^ | 4.24(0.42) | 207.50(14.94) | 4.68(0.54) |
| *ctl-1* | 177.00(22.99)^-^ | 4.84(0.06)^+^ | 198.40(8.96) | 5.71(0.15)^+^ | 120.20(58.98)^-^ | 4.17(0.18) | 73.00(17.21)^-^ | 4.77(0.15)^-^ |
| *cyp-37A1* | 301.00(18.06) | 4.46(0.03) | 149.00(14.41) | 5.78(0.19)^+^ | 311.40(11.06) | 4.44(0.05)^+^ | 298.60(58.54)^+^ | 5.42(0.22) |
| *dhs-28* | 129.60(53.53)^-^ | 5.99(0.16)^+^ | 46.50(23.38)^-^ | 5.34(0.46) | 138.33(45.63)^-^ | 5.51(0.22)^+^ | 30.00(20.92)^-^ | 5.80(1.08)^+^ |
| *dpy-14* | 53.60(41.51)^-^ | 5.44(0.36)^+^ | 16.60(15.26)^-^ | 5.29(0.21) | 38.40(18.50)^-^ | 4.93(0.13)^+^ | 0.60(0.55)^-^ | 6.33(0.58)^+^ |
| *dpy-17* | 125.83(15.87) | 4.90(1.12) | 144.83(45.08) | 4.93(0.39) | 173.00(113.0)^-^ | 4.19(0.42) | 142.83(137.8) | 4.19(0.46)^-^ |
| *elo-5* | 276.00(13.42) | 3.97(0.05)^-^ | 179.00(8.86) | 4.70(0.10)^-^ | 342.60(13.58) | 4.16(0.08) | 247.00(7.58) | 3.85(0.02)^-^ |
| *F55F3.3* | 198.00(7.11)^-^ | 4.19(0.11) | 118.20(56.23) | 5.27(0.46) | 31.20(4.66)^-^ | 4.26(0.10) | 72.40(15.01)^-^ | 5.91(0.26)^+^ |
| *fat-2* | 238.20(44.79) | 4.61(0.10) | 225.60(13.18) | 4.98(0.08) | 328.40(9.50) | 4.02(0.02) | 283.40(18.64)^+^ | 4.89(0.11) |
| *gei-7* | 299.20(26.99) | 4.54(0.20) | 186.67(56.70) | 5.18(0.34) | 302.33(40.83) | 4.31(0.22) | 200.67(85.45) | 4.36(0.29)^-^ |
| *gld-1* | 222.40(19.19)^-^ | 4.71(0.10)^+^ | 81.40(27.65)^-^ | 4.77(0.22) | 215.00(50.91)^-^ | 4.24(0.27) | 165.80(14.94) | 5.00(0.05) |
| *hsp-12.6* | 136.40(12.99)^-^ | 4.34(0.04) | 97.40(21.13) | 4.99(0.15) | 135.40(27.93)^-^ | 3.72(0.07)^-^ | 109.80(14.72)^-^ | 4.27(0.08)^-^ |
| *mtl-2* | 361.20(42.44) | 4.44(0.17) | 244.67(32.66)^+^ | 5.00(0.41) | 346.00(28.64) | 4.16(0.21) | 269.50(42.79) | 4.26(0.26)^-^ |
| *pab-2* | 300.20(11.65) | 4.01(0.03)^-^ | 100.20(58.38) | 4.04(0.30)^-^ | 304.00(8.12) | 3.94(0.14) | 279.40(16.82)^+^ | 4.84(0.10)^-^ |
| *rol-6* | 162.00(22.36) - | 4.74(0.27) | 200.60(27.44) | 6.43(0.23)^+^ | 211.60(23.57)^-^ | 4.72(0.02)^+^ | 257.60(18.39) | 5.91(0.03)^+^ |
| *sqt-2* | 233.80(8.11) | 5.01(0.04)^+^ | 180.60(12.14) | 5.25(0.10) | 339.00(12.57) | 4.44(0.03)^+^ | 218.00(107.1) | 4.20(0.12)^-^ |
| *Y57A10C.6* | 267.80(43.61) | 4.60(0.13) | 60.00(80.02)^-^ | 4.98(0.87) | 256.67(12.74)^-^ | 4.41(0.29) | 184.33(40.76) | 5.30(0.51) |
