# Supplementary material for: Caenorhabditis elegans Genomic Response to Soil Bacteria Predicts Environment-Specific Genetic Effects on Life History Traits
Source: PLoS Genet. 2009 Jun 5;5(6):e1000503. doi: 10.1371/journal.pgen.1000503 (PMC2684633; doi:10.1371/journal.pgen.1000503)
Supplement: Table S3 — qPCR validation results. We used quantitative reverse transcriptase real time PCR to validate the results of the microarray experiments. Three new biological replicates that were not used in the microarray experiments were used for validation. cDNA was synthesized for these RNA samples using a two-step iScript cDNASynthesis Kit (BioRad Laboratories) and these three replicate cDNA stocks for each bacterial environment were used for all subsequent tests. qPCR was performed with a Bio-Rad icycler (Bio-Rad Laboratories) using transcript specific primers. PCR primer sequences are available upon request. PCR reaction parameters were optimized as needed and housekeeping genes were used to standardize and calculate ΔCT values. From this ΔΔCT values were calculated and are shown in in the columns labeled qPCR. Microarray expression differences are shown for comparison. Melt curve analyses were performed to test for specific amplification. In all cases amplification was specific. (0.06 MB DOC) [file pgen.1000503.s003.doc]

**Supporting Table 3**: qPCR validation results

|  | *B. megaterium*  vs. E. coli | | *B. megaterium*  vs.  *M. luteus* | | *B. megaterium*  vs.  *Pseudomonas* | | *E. coli*  vs.  *Pseudomonas* | | *E. coli*  vs. M. luteus | | M. luteus vs.  *Pseudomonas* | |
| --- | --- | --- | --- | --- | --- | --- | --- | --- | --- | --- | --- | --- |
| Gene | Array | qPCR | Array | qPCR | Array | qPCR | Array | qPCR | Array | qPCR | Array | qPCR |
| *lys-4* | 1.814 | 2.01 | NS | NS | 4.9792 | 4.43 | 3.1652 | 3.03 | 1.8028 | 2.12 | 4.9681 | 5.31 |
| *ilys-3* | NS | NS | 1.7127 | 1.98 | 2.5388 | 2.12 | 2.2666 | 2.32 | 1.9848 | 1.88 | 4.2514 | 4.11 |
| *cpi-1* | 3.6191 | 4.03 | NS | NS | 2.8718 | 2.77 | NS | NS | 2.7359 | 2.53 | 1.9885 | 2.61 |
| *dpy-17* | 1.6517 | 1.72 | 1.1972 | 1.45 | NS | NS | NS | NS | NS | NS | NS | NS |
| *gei-7* | 1.8005 | 1.67 | 1.1629 | 1.28 | 1.8091 | 2.21 | NS | NS | NS | NS | NS | NS |
| *mtl-2* | 3.0595 | 2.89 | NS | NS | NS | NS | 2.1706 | 2.22 | NS | NS | NS | NS |
| *dhs-28* | NS | NS | 1.5478 | 1.67 | NS | NS | NS | NS | 1.9573 | 1.92 | 2.0024 | 2.53 |
| *Y57A10C.6* | NS | NS | 2.1269 | 2.03 | NS | NS | NS | NS | 2.6368 | 2.33 | 3.0965 | 3.62 |
| *act-4* | NS | NS | NS | NS | 1.8426 | 1.55 | 2.5454 | 2.13 | NS | NS | 2.0024 | 2.16 |
| *acdh-1* | NS | NS | 2.7392 | 2.56 | NS | NS | NS | NS | NS | NS | 2.7293 | 2.48 |
| *sodh-1* | 3.4823 | 3.06 | NS | NS | 2.8544 | 3.98 | NS | NS | 3.6534 | 5.31 | 3.0254 | 6.17 |
| *F41F3.3* | NS | NS | NS | NS | NS | NS | 2.7586 | 1.98 | NS | NS | 3.1077 | 2.68 |
