# Supplementary material for: Caenorhabditis elegans Genomic Response to Soil Bacteria Predicts Environment-Specific Genetic Effects on Life History Traits
Source: PLoS Genet. 2009 Jun 5;5(6):e1000503. doi: 10.1371/journal.pgen.1000503 (PMC2684633; doi:10.1371/journal.pgen.1000503)
Supplement: Table S4 — Regression statistics for generation time and brood size. Regression analysis was performed using Log2 transformed fold change values from the transcriptional profiling experiments as the independent variable and Log2 transformed fold change in generation time or brood size as the dependent variable. Correlation coefficients (r) and slope values for the best-fit lines are indicated. Additionally, P-values corresponding to tests of slope≠0 are shown. (0.04 MB DOC) [file pgen.1000503.s004.doc]

Supporting Table 4: Regression statistics for generation time and brood size

| Life history | Data set | r | Slope | Slope ≠ 0 *P*-value |
| --- | --- | --- | --- | --- |
| Log_2_(fold change generation time) | All | 0.057 | 0.054 | <0.0001 |
| Log_2_(fold change brood Size) | All | 0.34 | -0.051 | 0.5287 |
