# Supplementary material for: Caenorhabditis elegans Genomic Response to Soil Bacteria Predicts Environment-Specific Genetic Effects on Life History Traits
Source: PLoS Genet. 2009 Jun 5;5(6):e1000503. doi: 10.1371/journal.pgen.1000503 (PMC2684633; doi:10.1371/journal.pgen.1000503)
Supplement: Table S5 — Genes and alleles used for functional tests. List of 21 mutants used for functional tests and their predicted molecular functions and allele type are indicated. (0.05 MB DOC) [file pgen.1000503.s005.doc]

**Supporting Table 5**: Genes and alleles used for functional tests

| Gene | Allele | Predicted molecular function | Allele type |
| --- | --- | --- | --- |
| *acdh-1* | *ok1489* | Acyl-CoA dehydrogenase | Null |
| *C23H5.8* | *ok651* | Unknown function | Null |
| *cey-2* | *ok902* | Cold-shock/Y-box domain containing | Null |
| *cey-4* | *ok858* | Unknown function | Null |
| *cpi-1* | *ok1213* | Homolog of cysteine protease inhibitors (cystatins) | Null |
| *ctl-1* | *ok1242* | Cytosolic catalase | Null |
| *dhs-28* | *ok450* | 17-Beta-hydroxysteroid dehydrogenase 4 | Null |
| *dpy-14* | *e188* | Type III (alpha 1) collagen | Missense |
| *dpy-17* | *e1295* | Cuticle collagen | Loss-of-function, Not molecularly characterized |
| *elo-5* | *gk182* | PUFA elongase | Null |
| *cyp-37A1* | *ok673* | Cytochrome P450 | Null |
| *F55F3.3* | *ok1758* | Unknown function | Null |
| *fat-2* | *ok873* | Delta-12 fatty acyl desaturase | Null |
| *gei-7* | *ok531* | Predicted isocitrate lyase/malate synthase | Null |
| *gld-1* | *op236* | Meiotic cell cycle/oogenesis | Missense |
| *hsp-12.6* | *gk156* | Predicted heat shock protein | Null |
| *mtl-2* | *gk125* | Metallothionein | Null |
| *pab-2* | *ok1851* | Polyadenylate-binding protein | Null |
| *rol-6* | *e187* | Cuticle collagen | Loss-of-function, Not molecularly characterized |
| *sqt-2* | *sc108* | Cuticle collagen | Missense |
| *Y57A10C.6* | *ok693* | Predicted thiolase | Null |
