# Supplementary figures and images for: Caenorhabditis elegans Genomic Response to Soil Bacteria Predicts Environment-Specific Genetic Effects on Life History Traits
Source: PLoS Genet. 2009 Jun 5;5(6):e1000503. doi: 10.1371/journal.pgen.1000503 (PMC2684633; doi:10.1371/journal.pgen.1000503)

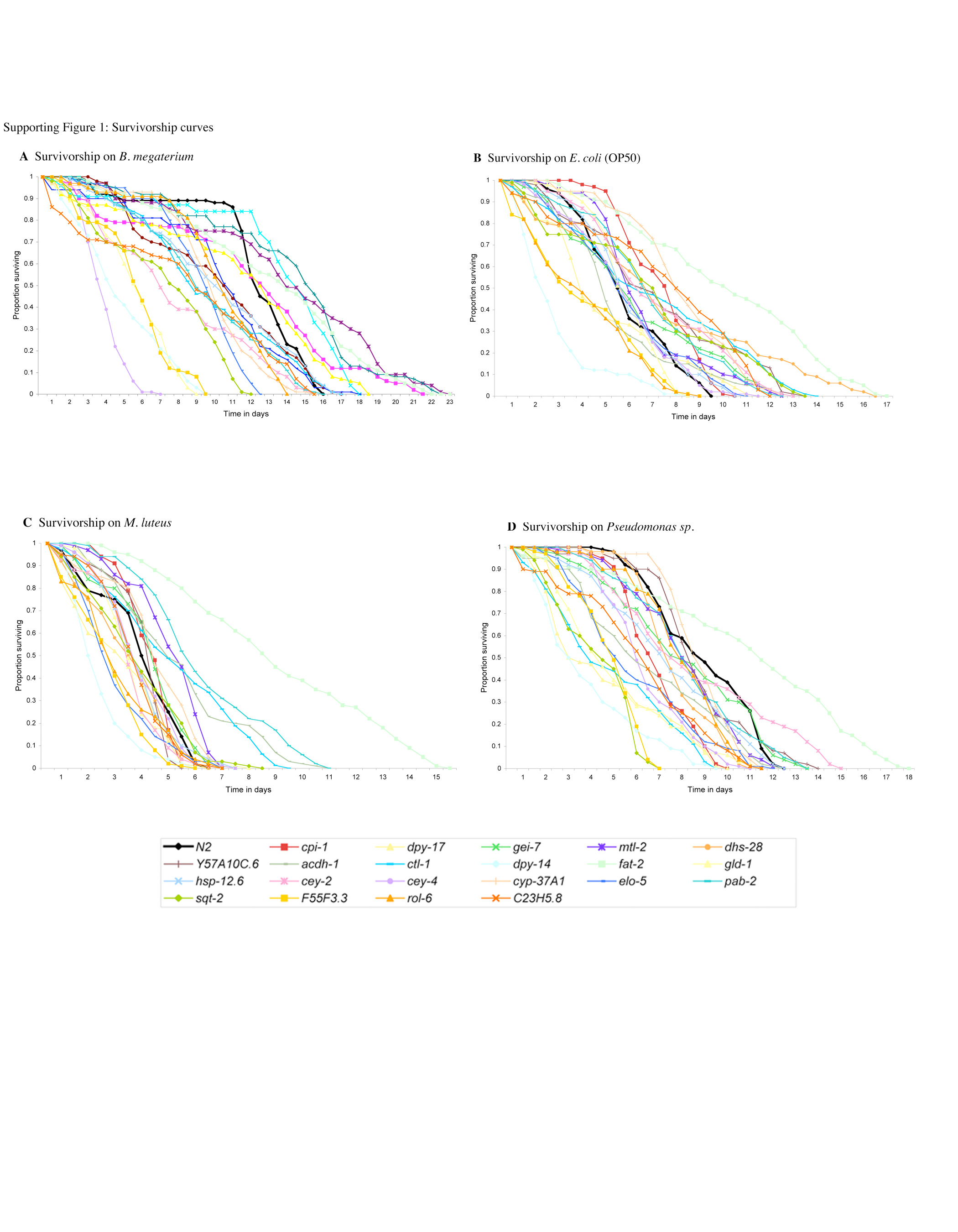

Supplement: Figure S1 — Survivorship curves. Survivorship curves are shown for N2 and mutant strains across time for bacterial environments: (A) B. megaterium (B) E. coli OP50 (C) M. luteus (D) Pseudomonas sp. (1.09 MB TIF) [file pgen.1000503.s006.tif]

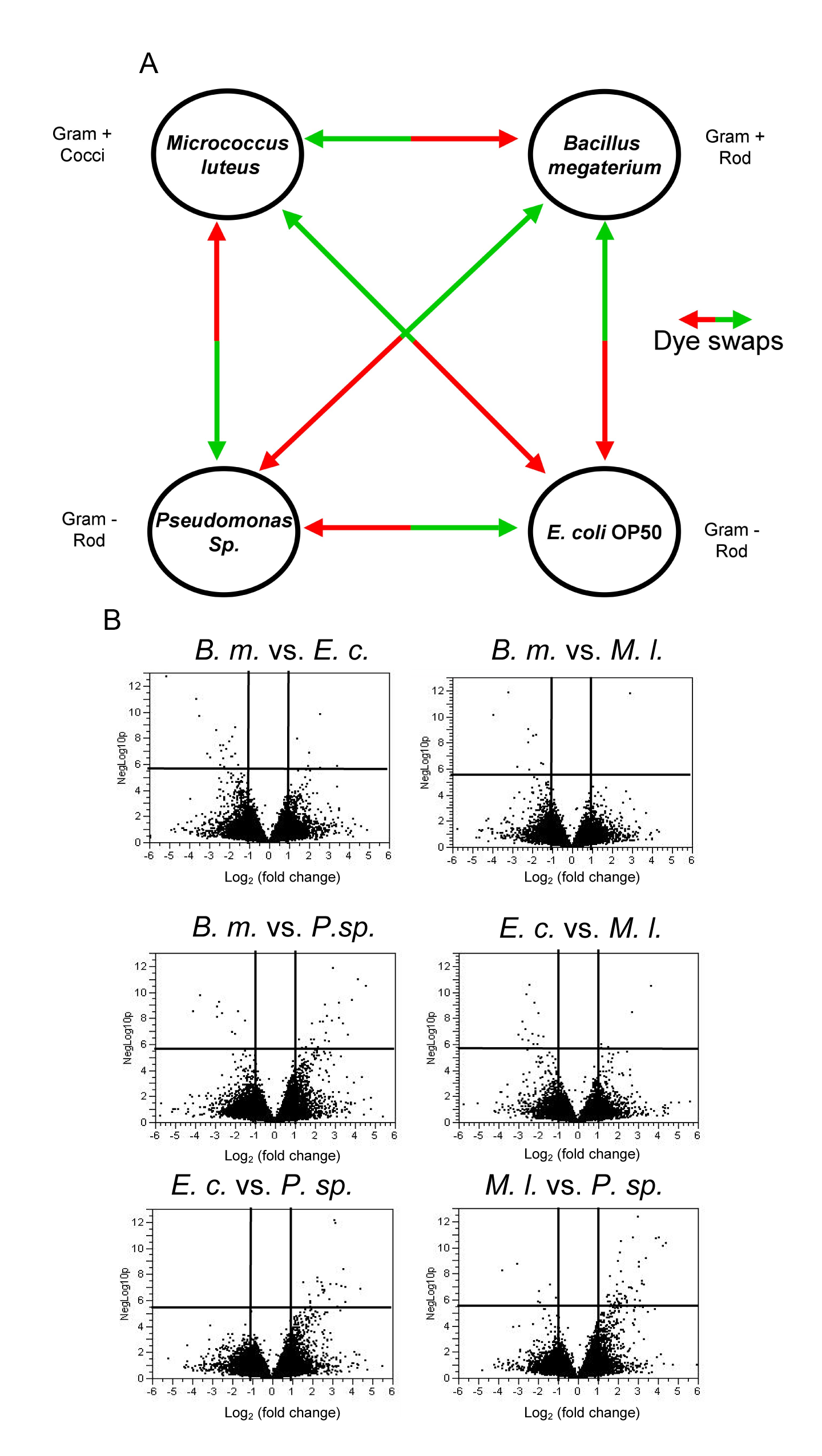

Supplement: Figure S2 — Microarray experimental design and analysis. (A) The experimental design for the microarray comparisons made is shown. All pairwise comparisons of adult C. elegans in the four bacterial environments were made in a factorial design. Six biological replicates were used and dye-swaps were preformed every other replicate. (B) Volcano plots are shown for each microarray comparison. For each, −log10(p-value) is plotted on the Y-axis and log2(fold change) is plotted on the X-axis. Data points represent the response of all the genes present on the microarrays used, with each point representing a single gene. Points above the horizontal line are significant at the false discovery rate q<0.01. The two vertical lines show 2 fold up or down regulation in the treatment (relative to the second listed bacteria in graph titles). (0.75 MB TIF) [file pgen.1000503.s007.tif]

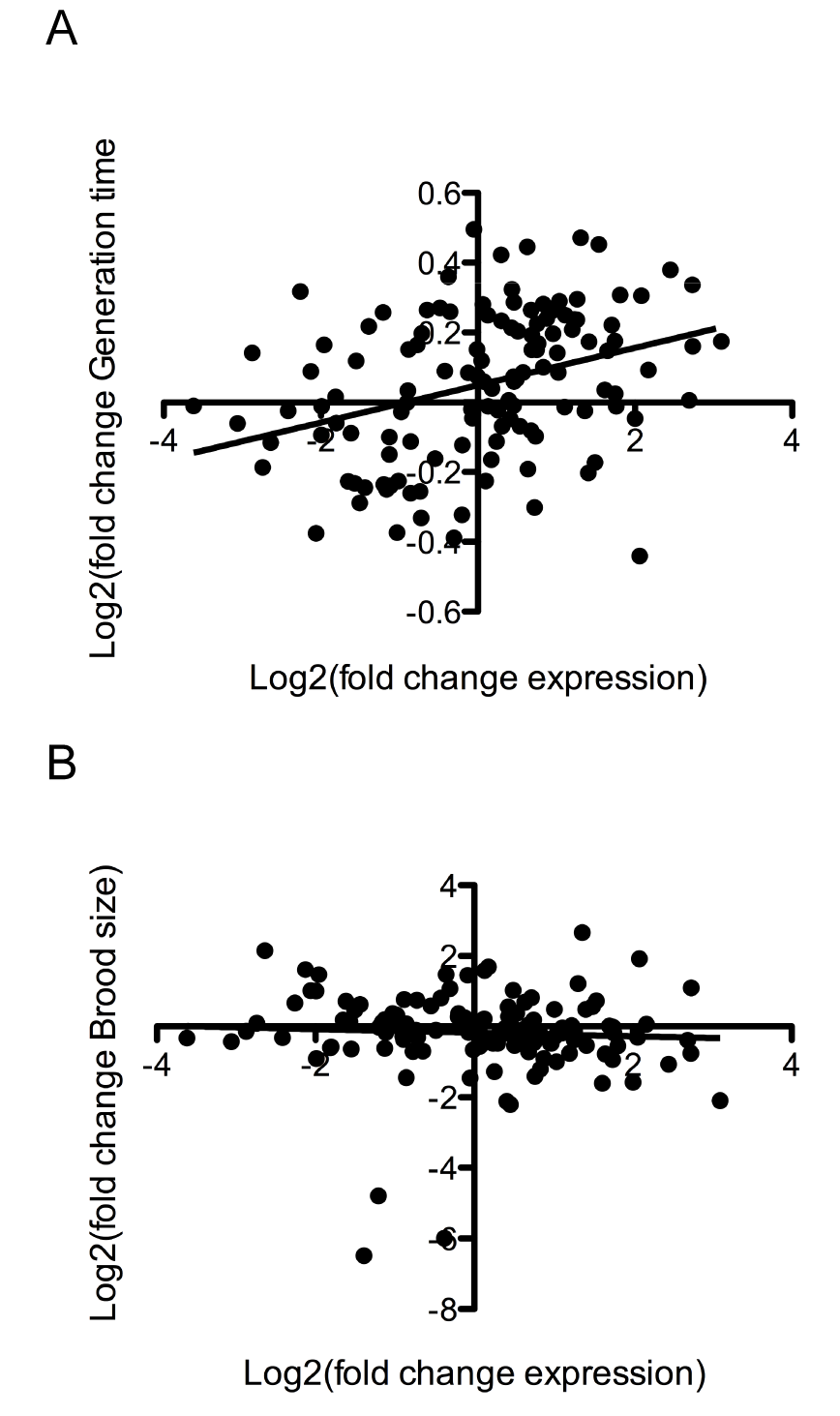

Supplement: Figure S3 — Regression analysis of generation time and brood size. Linear Regressions were performed using Log2 transformed fold change in gene expression from microarray experiments as the independent variable and Log2 transformed fold change in (A) Generation time or (B) Brood size in bacterial comparisons of mutant life history traits. (0.18 MB TIF) [file pgen.1000503.s008.tif]

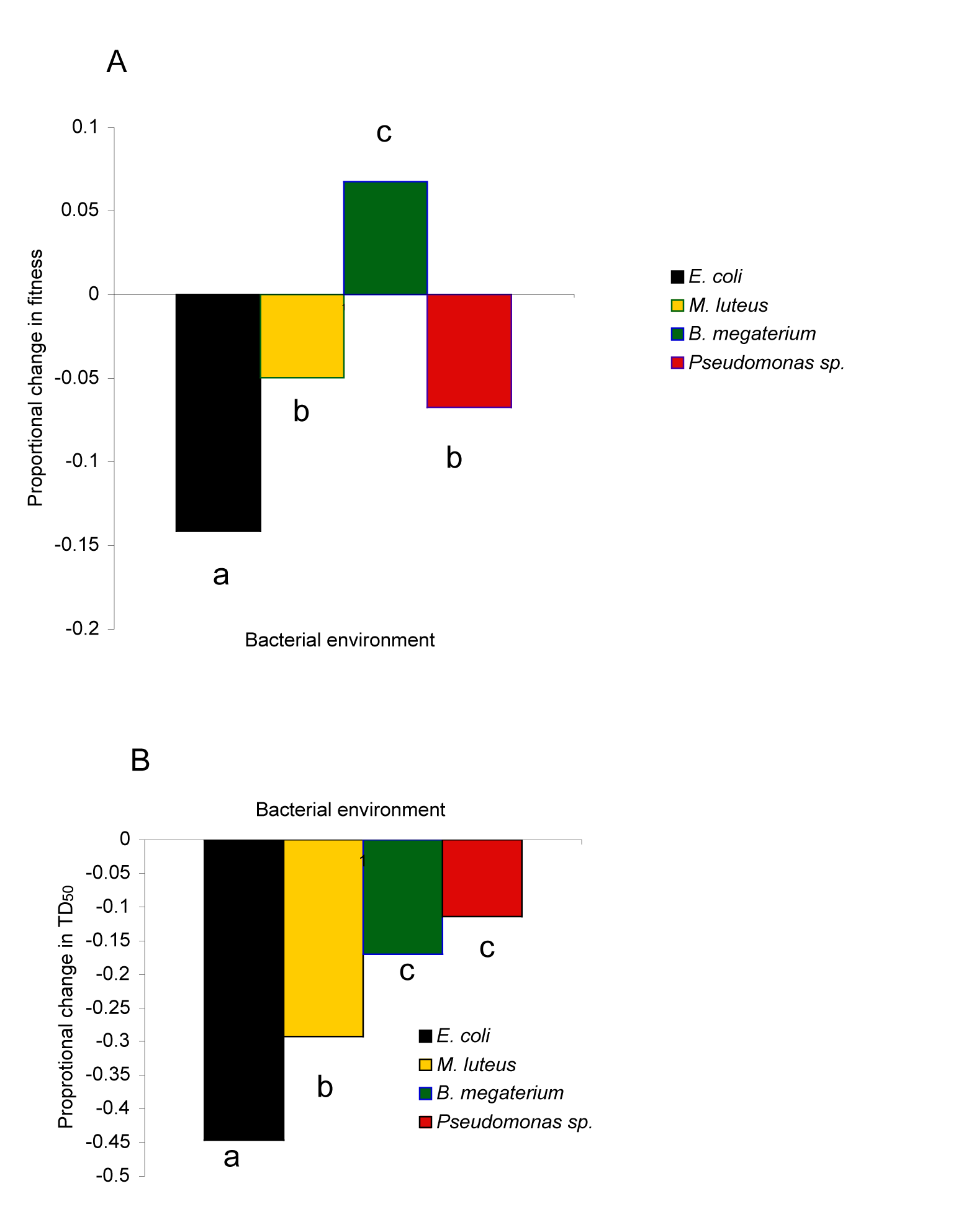

Supplement: Figure S4 — (A) Proportional changes in hsp-12.6 fitness were calculated as (μN2−μhsp-12.6)/μN2 by bacterial environment to make relative to wild type. (B) Proportional changes in rol-6 longevity (measured as TD50) relative to wild type calculated as (μN2−μrol- 6)/μN2 by bacterial environment. Letters indicate significantly different means (P>0.05 by ANOVA). (0.13 MB TIF) [file pgen.1000503.s009.tif]
